# Potential of Banana peel extract powder as a promising alternative to antibiotics for treating *Salmonella* Gallinarum infection in broiler chicks

**DOI:** 10.1016/j.psj.2025.106087

**Published:** 2025-11-10

**Authors:** Gulbeena Saleem, Nabiha Fatima, Maryam Tariq, Asad Ullah, Bushra N. Khan, Sadia Chaman, Mostafa A. Abdel-Maksoud, Abdulaziz Alamri, Aljawharah F. Alabbad, Fatma Sh. Kalmosh

**Affiliations:** a Department of Pathology, University of Veterinary and Animal Sciences, Lahore, Pakistan; b Institute of Zoology, University of the Punjab, Lahore, Pakistan; c Institute of Pharmaceutical Sciences, University of Veterinary and Animal Sciences, Lahore, Pakistan; d Research Chair of Biomedical applications of nanomaterials, Biochemistry Department, College of Science, King Saud University, Saudi Arabia; e Biochemistry department- College of Science- King Saud University, Riyadh, Saudi Arabia; f Botany and Microbiology department, College of Science, King Saud University, Saudi Arabia; g State Key Laboratory for Biology of Plant Diseases and Insect Pests, Institute of Plant Protection, Chinese Academy of Agricultural Sciences, Beijing, China

**Keywords:** Banana peel extract powder (BPP), *Salmonella* Gallinarum, Gene expression, Growth performance and broiler chicken

## Abstract

With the global rise in antibiotic resistance, exploring plants for their potential as safe and natural alternatives to antimicrobials in poultry has become increasingly important. The present study aimed to evaluate the ethnoveterinary supplementation of banana peel extract powder (BPP) against *Salmonella enterica* subspecies *enterica* serovar Gallinarum biotype Gallinarum (*Salmonella* Gallinarum), focusing on two key immune-related genes (IL6 and MHC class IIβ), gut morphology, and growth performance of broiler chickens experimentally challenged with *Salmonella* Gallinarum. A total of 180 day-old Hubbard Classic male chicks were divided into five treatment groups; a non-challenge control (NC), *Salmonella* Gallinarum infected birds (SGI) and three treatments receiving 2 % (BPP-1), 4 % (BPP-2) and Ciprofloxacin (CIPRO) along with *Salmonella* Gallinarum challenge. Each treatment was replicated in triplicate, with 36 birds per group. The chicks in challenge groups were orally inoculated on day 7 with 1 mL of 2 × 10⁸ CFU *Salmonella* Gallinarum, while chicks in NC group received 1 mL of sterile broth. Liver and caecal samples were collected on days 7, 10, 14 and 21 for gene expression analysis of IL6 and MHC class IIβ using qPCR. Histopathological examination of the spleen, liver and caeca was performed on days 10, 14 and 21. Feed intake, feed conversion ratio (FCR) and weight gain were monitored throughout the experiment. Challenge with *Salmonella* Gallinarum (SGI group) resulted in significantly higher IL-6 expression and lower MHC class IIβ expression (*p* < 0.01) in both liver and caeca. In contrast, BPP-1 and BPP-2 supplementation led to lower IL-6 with elevated MHC class IIβ expression, indicating a more regulated immune response. In conclusion, dietary supplementation with banana peel, particularly 4 % modulates immune related gene expression (IL6 and MHC class IIβ), improves gut health and growth performance against *Salmonella* Gallinarum infection in broiler chicks.

## Introduction

*Salmonella enterica* subspecies *enterica* serovar Gallinarum biotype Gallinarum (*Salmonella* gallinarum) is the etiological agent of fowl typhoid, a disease that poses a significant economic threat to the poultry industry, with an estimated global prevalence of 8.54 %. The highest incidence has been reported in developing regions, particularly Asia (17.31 %) (Shivaprasad, 2000). The disease affects both broilers and layers and is characterized by an acute systemic infection that may occur at any stage of production (Kwon et al., 2010; Zhou et al., 2022; Aganja et al., 2025). High mortality and decreased productivity in *Salmonella* Gallinarum infected flocks result in substantial economic losses for poultry producers (Farhat et al., 2023). Current control strategies primarily rely on strict biosecurity measures, vaccination, and the use of antibiotic (Totton et al., 2012; Naumovska et al., 2025). However, the extensive prophylactic and therapeutic use of antimicrobials has led to the emergence of genetically mutable *Salmonella* Gallinarum strains (Seo et al., 2019; Duysak et al., 2023). As resistant bacteria can be transmitted from poultry to humans through handling or consumption of contaminated meat. There is an urgent need to explore natural alternatives to antibiotics, such as plant-derived products with potential antimicrobial and immunomodulatory properties.

Many plants exhibit antimicrobial potential due to bioactive phytochemical compounds produced during secondary metabolism (Al-Mnaser et al., 2022; Zhang et al., 2025). The mode of action and target sites of these phytochemicals vary at both the cellular and molecular levels (Almuzaini, 2023). At the cellular level, phytochemicals disrupt the structural components such as the cell wall, cell membrane, capsule and mitochondria ultimately leading to the death of the pathogenic microorganism (Khare et al., 2021). At the molecular level, these phytochemicals interact with key macromolecules, rendering them non-functional. The antimicrobial activities of these secondary metabolites, however are concentration dependent (Tsuchiya, 2015). When incorporated into conventional poultry diets, plant extracts can improve the intestinal microflora and overall gut health (Al-Mnaser et al., 2022). Despite this potential, the growth-promoting and antibacterial effects of many plants and their parts remain largely unexplored (Lillehoj et al., 2018).

Banana (*Musa sapientum* Linn *Musa paradisiaca*) is among the most produced fruit globally, with annual production exceeding 7 million tons. It serves as a rich source of starch, amino acids, fatty acids and potassium and is also valued for its abundant phytochemical compounds (Imam and Akter, 2011). Banana peel (BP), a major by-product, constitutes approximately 30 % to 40 % of the total weight of the ripe banana (Zou et al., 2022). The presence of various bioactive compounds in BP, particularly dietary fiber and phenolic compounds, indicates its potential biological activity. Its antioxidant activity is primarily attributed to compounds such as gallocatechins and dopamine (Chueh et al., 2019; Dumorné et al., 2020). Several studies have also demonstrated its antibacterial properties (Hanafy et al., 2021; Al-Jassani, 2023).

Various bioactive phytochemical constituents such as tannins, alkaloids, flavonoids, saponins and anthraquinones have shown promising antibacterial effects against different bacteria, predominantly *Escherichia coli, Staphylococcus aureus* and *Salmonella* species (Nascimento et al*.*, 2000; Totton et al., 2012). Beyond antibacterial activity, saponins and anthraquinones also possess anti-inflammatory properties (Hashim et al., 2023). Recent studies have shown that BP effectively inhibits spoilage bacteria in aquatic products (Ansari et al., 2023; Meng et al., 2025). Banana flour has also been used in broilers as a useful and low cost alternative feed ingredient for animal feed (Dumorné et al., 2020). When incorporated into feed or water, BPP has been reported to mitigate infection and improve gut health in poultry (Araya et al., 2021).

While previous studies have demonstrated the antioxidant, antimicrobial, and anti-inflammatory properties of BP in non-avian models and against common poultry pathogens, its effects on *Salmonella* Gallinarum infection remain unexplored. Therefore, this study aimed to evaluate the effects of dietary BPP supplementation on the expression of key immune-related genes (IL6 and MHC Class IIβ), intestinal histopathology, and growth performance in broilers chickens.

## Materials and methods

### Collection and authentication of banana peel

Fresh ripe bananas (yellowish green color) were purchased from local market. Banana peels were removed, washed, and sieved for 30 min followed by drying in an oven at 60°C for 8 hrs. Dried BP was powdered and stored in airtight containers. The dried peel was ground to fine powder by a pulverizing machine (VM-SS3HP). Banana peel samples were authenticated by a taxonomist at Government College University Lahore and voucher receipt (GC.Herb.Bot.3421) was collected. Proximate analysis was performed on powdered peel (USP 2003). Two experimental diets were prepared one with 20 mg/kg and 40 mg/kg of BPP in broiler feed.

### Extraction of banana peel

Extraction of banana peel was done according to the method as described by Mazumder (2024) with slight modifications. Briefly, powdered banana peel (50 g) was soaked in 250 mL methanol (Thermo Fisher Scientific, Waltham, MA) in a round bottom flask for 24 h. The extract was then filtered by using Whatman filter paper No 1. The filtrate was dried and the dried powder was stored in bottled for further use.

### Phytochemical analysis

***Qualitative screening.*** The presence of steroids, alkaloids, glycosides, tannins, flavonoids, terpenoids, and phenols in BPP was determined according to the methods as described by (Kibria et al., 2019; Nasir et al., 2019).

***Quantitative analysis.*** Total polyphenol content (TPC) was quantified with Gallic acid as the standard, according to the method as described by (Slinkard and Singleton, 1977). For total flavonoid content (TFC), quercetin was used as standard using the method (Chang et al., 2002), whereas Alkaloid content were estimated according to standard protocols (Jain et al., 2021).

### Gas chromatography-mass spectrometry (GC-MS) analysis

Gas Chromatography-Mass Spectrometry (GC-MS) analysis was performed using an Agilent Technologies GC-MS system (Santa Clara, CA) with a 6850 Network GC system and 5973-mass selective detector. The identification of the bio compounds from BPP was performed and subsequently compared with the NIST 05 Mass Spectral Library and published spectra (EL-Kamali et al., 2009).

### High-performance liquid chromatography (HPLC)

Identification of phenolic acids in methanolic extract of banana peel was done by using the method described by Zhang et al. (2019). Briefly, banana peel extract (20 µL) was filtered and injected into HPLC system consisting of Agilent G1311A pump, Agilent G1315B diode array detector and Agilent G13290A automatic sample. The column used was C18 with dimensions and particle size of 250 mm × 4.6 mm and 5 µm respectively. The mobile phase was a combination of two solvent systems; 0.1 M acetonitrile and phosphoric acid (17:83) and 0.1 % acetonitrile and formic acid (15:85). The set flow rate was 1 mL per min and Wavelength of the detector were at 275 and 210 nm respectively for each system. Gallic acid (GA), Sinapic acid, and Ferulic acid were used as a standards in HPLC. The phenolic compounds were identified on the basis of the retention time (RT).

### In vitro efficacy of banana peel powder against *Salmonella* Gallinarum

Antibacterial activity of BPP was evaluated by using well diffusion assay according to the method as described by (Sharma and Chattopadhyay, 2015). Using a sterile swab, approximately 100 µL of freshly grown *Salmonella* Gallinarum culture (1 × 10^8^ CFU/mL) was streaked on the Mueller-Hinton agar plates (MHA) (Oxoid, Basingstoke, Hampshire, UK). Using a sterile corkborer, 6 mm wells were punched on MHA plates and sealed using molten agar. Aseptically, 25 μL of BPP at different concentration (50, 100, 200 and 400 mg) were added to these wells. Ciprofloxacin (commonly used antibiotic against *Salmonella* Gallinarum was taken as a standard antibiotic) to be used as positive control. The plates were incubated at 37°C for 24 h. Following incubation, zones of *Salmonella* Gallinarum growth inhibition were measured in millimeters (mm) and each test was performed in triplicates.

### Determination of minimum inhibitory concentration (MIC)

The minimum inhibitory concentration (MIC) of BPP against *Salmonella* Gallinarum was determined using the broth microdilution method according to the procedure as described by (Angane et al., 2022). Stock solution of BPP was prepared a concentration of 2000 μg/mL. Serial dilution of the stock solution was prepared in Nutrient Broth (Oxoid, Basingstoke, Hampshire, UK) in 96 well microplates. Then 100μl of a *Salmonella* Gallinarum suspension of approximately 10^8^ CFU/mL prepared from 24 h culture plate and approximately 1000μl of *Salmonella* Gallinarum suspension was inoculated into each plate. The plates were incubated at 37°C for 24 h. After incubation the well which has no turbidity was considered as MIC.

### Experimental design

All the experimental procedures were approved by the Animal Ethics Committee of the university. A total of 180 day-old male Hubbard Classic chicks were divided into 5 treatments with 3 replicate pens, each having 12 birds. The treatments included: a non-challenge control (NC) receiving 1 mL of sterile Brain Heart Infusion broth (BHI) (Oxoid, Basingstoke, Hampshire, UK). *Salmonella* Gallinarum infected birds (SGI) receiving the challenge only; BPP-1 and BPP-2 receiving 2 % (20 mg/kg) and 4 % (40 mg/kg) of BPP respectively from day 3 until day 21 of age along with SG challenge, and a CIPRO group receiving Ciprofloxacin (10 mg/kg) for 3 consecutive days post-challenge. Fecal samples were analyzed for bacterial analysis and serum plate agglutination tests using *Salmonella* Gallinarum antigen to make sure that all the chicks were Salmonella free. All the birds were fed the same starter diet and grower diet throughout the experiment. The birds were given water and feed *ad libitum*. During the experimental trial, 23 h light and 1 h darkness were provided with temperature range from 33°C and 30°C during the first 14 days following gradual reduction to 21°C by day 21. All the birds were vaccinated against Newcastle disease and IBD as per routine local vaccination practices.

### *Salmonella* Gallinarum challenge

*Salmonella* Gallinarum stock, used previously (Liaqat et al., 2021), was thawed and inoculated into Tetrathionate Broth (TTB) (Oxoid, Basingstoke, Hampshire, UK) followed by incubation at 42°C for 48 h. The incubated culture was then streaked onto Salmonella Shigella (SS) Agar (Oxoid, Basingstoke, Hampshire, UK) and Brilliant Green Agar (BGA) (Oxoid, Basingstoke, Hampshire, UK) and subsequently examined for typical Salmonella colonies. An individual bacterial colony was transferred into BHI and incubated in a shaking incubator (160 rpm) at 42°C overnight. Following incubation, serial dilutions were made in normal saline (0.9 %) to calculate CFU. On day 7, birds in challenge treatment groups were challenged with 2 × 10⁸ CFU of *Salmonella* Gallinarum via oral gavage of 1 mL of BHI broth.

### Sample collection and analysis

Birds were monitored twice daily for signs and symptoms of fowl typhoid, as described by Saleem et al. (2022) untill day 21. On days 7 (pre-challenge), 10, 14, and 21 (post-challenge), two birds from each replicate pen were humanely euthanized by cervical dislocation. A comprehensive and systematic postmortem examination was performed to identify the lesions typical of Salmonella infection, primarily characterized by bronzed discoloration of the liver, intestinal hemorrhages, splenomegaly, and vent pasting (Saleem et al., 2022). The Spleen, liver, and caeca were collected for histopathological examination while only the liver and caeca were used for RNA isolation and subsequent gene expression analysis via qPCR.

***Gene expression analysis****.* Following necropsy, tissue samples from the caeca and liver were aseptically collected to evaluate expression levels of IL-6 (pro-inflammatory) and MHC class IIβ genes (immunity related), and stored in RNAlater® solution (Thermo Fisher Scientific, Waltham, MA) at −80°C until further processing.

Total RNA was isolated from caeca and liver tissue using TRIzol® Reagent (Rio et al., 2010) according to manufacturer's recommendation. The quality of RNA was checked by using a NanoDrop™ 2000 spectrophotometer (Thermo Fisher Scientific, Waltham, MA), and Bioanalyzer (Agilent Technologies, Santa Clara, CA) by measuring optical density at 260 nm and 280 nm. Samples with an RNA Integrity Number (RIN) above 8.0 were utilized for subsequent analyses. For the synthesis of cDNA, 1 μg of RNA was reverse-transcribed using the High-Capacity cDNA Reverse Transcription Kit (Thermo Fisher Scientific, Waltham, MA) following manufacturer’s protocol.

Gene expression levels for IL-6 and MHC class IIβ were quantified using previously validated primers (Zhang et al., 2025) and quantified by quantitative real-time PCR (qPCR) (Applied Biosystems™ Waltham, MA) using SYBR green dye. GAPDH was used as an internal control and negative control was also set. Primer sequences and gene accession numbers are provided in Table 3. Relative gene expression was determined using the comparative CT method 2^^-ΔΔCt^ normalized to endogenous reference gene relative to calibrator (Livak and Schmittgen, 2001).

***Enumeration of Salmonella* Gallinarum***.* Following postmortem examination, caecal digesta and liver samples were aseptically removed for determination of *Salmonella* Gallinarum on days 7 (pre-challenge), 10, 14, and 21 (post-challenge). Samples were weighed and plated on Tryptic Soy Broth (TSB) for pre-enrichment and incubated for 24 hours at 37°C. After pre-enrichment, colonies were transferred to Rappaport Vassiliadis Soya broth (RVS) (Oxoid, Basingstoke, Hampshire, UK) for selective enrichment and incubated at 37°C for 24 h. Afterwards, a loopful of RVS streaked on SS agar and BGA. After streaking, the plates were incubated at 37°C for 24 h and subsequently observed for bacterial colonies to calculate CFU of *Salmonella* Gallinarum in digesta and liver samples (Zhang et al., 2025). The results were expressed as log_10_ CFU/g. Liver and intestines were also collected and stored in 10 % neutral buffered formalin for histopathological studies.

***Histopathological Assessment Analysis****.* Caecal, spleen and liver tissue samples from two birds from each replicate were collected at the end of experiment (day 21). Tissue samples were washed with sterile saline (0.9 %) solution to remove the digesta and preserved in 10 % neutral buffered formalin, followed by processing by paraffin embedding technique (Saleem, 2013). The paraffin blocks were cut at 4 µm thickness and stained with hematoxylin and Eosin (H and E). The slides were examined under a light microscope with a digital imaging system (Olympus, Corporation, Tokyo, Japan) to determine histopathological changes associated with fowl typhoid (Zhang et al., 2025).

***Growth performance****.* Upon arrival the body weights (BW) of birds was recorded on individual basis. Feed intake, body weight gain and feed conversion ratio (FCR) were sequentially noted on 7, 14 and 21 days. FCR was calculated according to the following formula in order to assess the growth performance of birds.

$$FCR=\;\frac{Feed\;given\;\left(Kg\right)}{Animal\;weight\;gain\;\left(Kg\right)}$$

### Statistical analysis

Relative gene expression for qPCR data, was calculated using the 2^-ΔΔCt method. Differences among the groups were evaluated using one-way analysis of variance (ANOVA) using GenStat 11 for Windows (VSN International Ltd, Hemel Hempstead, UK). Each replicate pen and block was included as random effects in the statistical model. Due to the nonnormal distribution of bacterial count data (CFU) and the presence of zero values, data were log₁₀-transformed using the formula log₁₀ (CFU + 1). The constant 1 was added to all values to allow logarithmic transformation of zero counts. Normality of transformed data was assessed using the Shapiro-Wilk test. Differences among treatments were considered statistically significant at *p* < 0.05. Where significant differences occurred, Bonferroni's test was conducted for post-hoc comparisons to determine significant differences between specific groups. Results are presented as means ± standard error of the mean (SEM).

## Results

The proximate analysis of BPP showed the sample contained 4 % moisture, 5.2 % total ash, 2.1 % water soluble ash, 1.1 % acid insoluble ash, 2.2 % water soluble extractives and 17.4 % alcohol soluble extractives. Phytochemical analysis (qualitative and quantitative) of BPP showed the presence of alkaloids, flavonoids and tannins (Table 1) and a percentage yield of 11.372. Total polyphenol content were present in significantly higher concentrations compared to total flavonoids and alkaloids (Table 2). The GC-MS analysis identified various compounds of banana peel powder and their respective biological activity referenced with supporting literature along with their diverse therapeutic potentials (Table 6). Based on RT, BPP was positive for gallic acid, fumaric acid and caffeic acid on high-performance liquid chromatography. *In- vitro* analysis of antibacterial activity BPP showed varying inhibition patterns with Ciprofloxacin. Minimum inhibitory concentration (MIC) was found to be 6.25 mg/mL against *Salmonella* Gallinarum.

**Table 1:** Phytochemical composition of banana peel crude powder.

Phytochemicals	Banana peel (Crude powder)
Alkaloids	+
Glycosides	-
Tannins	+
Flavonoids	+
Terpenoids	-
Carbohydrates	+
Proteins	+
Fats and fixed oils	-

+: present**; -** Absent.

**Table 2:** Quantitative phytochemical analysis of banana peel crude powder.

Total flavonoid content (QE mg/g)	Total polyphenol Content (GAE mg/g)	Total Alkaloids (%)	Total protein Content (%)
11.2 ± 0.1	18.7 ± 0.3	2.4 ± 0.3	2.2 ± 0.2

**Table 3:** Primer Sequence and gene accession number.

Gene	Chicken Primer Sequence (5′→3′)	Accession number
IL-6	F:AGCTGGAGTCACAGAAGGAGT R: GCGCAGAATGAGATGAGTTG	NM_204628.1
MHC-IIβ	F:CTGGACCTGGGAGACATCAC R: CAGGTAGTTGTGGGCGTGTT	XM_040696135.1

**Fig. 1:**
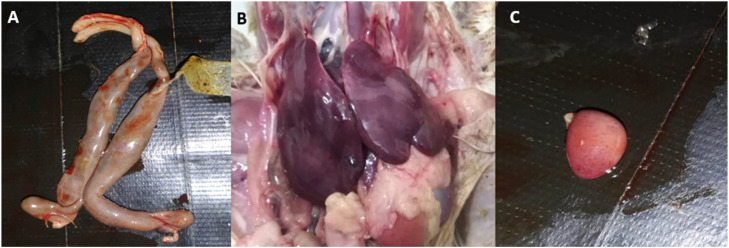
**Gross examination of lesions in broiler chicks experimentally challenged with *Salmonella* Gallinarum.** A; enteritis and ballooning of caeca, B; hepatitis and bronze discoloration of liver and C; multifocal hemorrhages on spleen.

Following experimental challenge, daily observation of birds for clinical manifestation of symptoms revealed that about ∼20 % of birds in SGI showed symptoms like ruffled feathers, huddling, dehydration and very few birds having yellowish-white diarrhea, consistent with fowl typhoid. None of the birds showed any signs of infection on day 7. Only two birds died during the experiment that was before the challenge and due to congestive heart failure. Postmortem examination on days 10, 14 and 21 showed that bronzed discoloration of the liver and hemorrhages in liver were the most common findings (Fig. 1B). Hemorrhages in spleen and ballooning of caeca were seen only in SGI group (Fig. 1A and 1C) All these findings indicate the moderate nature of challenge. Comparatively, BPP supplemented birds (2 % and 4 %) had lower number of birds with pathological lesions compared to the SGI group. Whereas, BPP-2 group, only two birds showed lesions of mild liver hemorrhages and three birds showed enteritis. These lesions were more pronounced on days 10 and 14. Additionally, a very mild degree of enteritis was observed in these birds. Ciprofloxacin treated group had the least pathological changes amongst all experimentally challenged birds. Control groups did not have any obvious pathological lesions in any of the birds on any of the days observed.

### Gene expression analysis

**Fig. 2:**
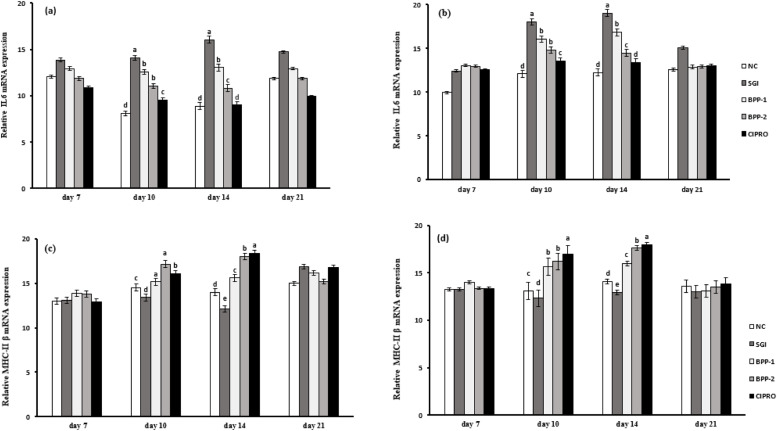
**Effects of banana peel extract powder supplementation on gene expression levels of Il-6 and MHC class IIβ in caeca and liver tissues of broiler chicks experimentally challenged with *Salmonella* Gallinarum.** (a) IL-6 gene expression in caeca, (b) IL-6 gene expression in liver, (c) MHC class IIβ gene expression in caeca and (d) MHC class IIβ in liver. (NC) Non-challenge control, (SGI) group challenged with Salmonella Gallinarum infection, (BPP-1) Salmonella Gallinarum challenge group supplemented with 2 % Banana peel extract powder, (BPP-2) Salmonella Gallinarum challenge group fed with 4 % Banana peel extract powder and (CIPRO) Salmonella Gallinarum group given ciprofloxacin treatment. Various values are shown as means and SEM is represented by vertical bars. Different alphabets (^a,b,c,bc and d^) show statistically significant difference for the individual days across various groups. The set level of significance was 0.05.

On day 7, no significant differences in the expression levels of either IL-6 were observed among treatment groups in both caeca (*P* = 0.953), and liver tissues (*P* = 0.61). A marked upregulation of IL-6 were observed in the SGI group on days 10 (*P* < 0.001) and 14 (*P* < 0.001) in both tissues, with peak expression on 14^th^ day compared to the NC group. Supplementation with BPP downregulated IL-6 expression, particularly BPP-2 showing significantly lower IL-6 expression than BPP-1 in both tissues on both days (10 and 14). Similarly, the CIPRO group showed significant downregulation of IL-6 on these days, indicating its ability to reduce inflammation. By day 21, IL-6 expression levels showed no significant differences among groups (*P* = 0.888) (Figs. 2a and 2b). On day 7, MHC class IIβ expression did not differ significantly among treatment groups in either caeca (*P* = 0.97) or liver (*P* = 0.76) (Figs. 2c and 2d). However, on day 10, SGI group had a marked downregulation of the MHC class IIβ gene in both tissues (*P* < 0.01) compared to the NC group. This downregulation intensified further by day 14 (*P* < 0.01), suggesting an impaired antigen presentation in response to bacterial challenge. Supplementation with BPP, resulted in increased expression of MHC class IIβ in the caeca, compared to the SGI group but lower than in the NC group (*P* < 0.05). A similar trend was observed in the liver; however, the upregulation of MHC class IIβ was comparatively lower. Among all treatments, BPP-2 showed the most favorable immune response (*P* < 0.05) in both tissues when compared to the NC. By day 21, in the caeca, MHC class IIβ expression remained lower in the SGI groups relative to the NC group. In contrast, BPP supplementation significantly enhanced MHC class IIβ expression (*P* < 0.05). In the liver, however, no significant differences in MHC class IIβ expression were detected across groups (*P* = 0.99).

### Enumeration of *Salmonella* Gallinarum

In liver tissue, significant differences (*P* < 0.05) were observed among treatment groups on 10th day. The SGI group had the highest *Salmonella* Gallinarum counts compared to all other groups (*P* = 0.026), indicative of the clinical phase of infection due to successful bacterial colonization (Table 4). In contrast, broiler chicks supplemented with BPP had a significant reduction in *Salmonella* Gallinarum counts, reflective of its immunomodulatory and growth-promoting efficacy. Furthermore, birds in the BPP-2 had lower *Salmonella* Gallinarum counts than BPP-1 and SGI (*P* = 0.026), indicative of a dose-dependent antibacterial effect of BPP. However, no significant differences were observed in *Salmonella* Gallinarum counts among the groups on day 14 (*P* = 0.259) and day 21 (*P* = 0.261).

**Table 4:** Effect of Banana peel extract powder supplementation on *Salmonella* Gallinarum counts (liver and ceacal digesta) of broiler chickens experimentally challenged with Salmonella Gallinarum (CFU/g).

GROUP	Liver (CFU/g)			Caecal digesta (CFU/g)		
	Day 10	Day 14	Day 21	Day 10	Day 14	Day 21
NC	0^a^	0	0	0^a^	0	0^a^
SGI	3.59^b^	1.98	2.25	2.42^b^	1.94	3.24^b^
BPP-1	2.94^b^	1.93	1.72	0.97^b^	0.96	0.77^a^
BPP-2	1.79^b^	1.53	1.52	0.77^b^	0.85	0.66^a^
CIPRO	1.88^b^	0.72	1.1	0.26^b^	0	0^a^
p-value	0.026	0.259	0.261	0.032	0.092	0.002
SEM	0.751	0.718	0.713	0.533	0.536	0.55

NC = Non-challenge control group; SGI = *Salmonella* Gallinarum challenge group; BPP-1 = group fed basic diet + 2 % banana peel extract powder + *Salmonella* Gallinarum challenge; BPP-2= group fed basic diet + 4 % banana peel extract powder with *Salmonella* Gallinarum challenge; CIPRO= Ciprofloxacin with *Salmonella* Gallinarum challenge. Values are represented as means and SEM;.

a,b Means in the same column with different superscripts are significant SEM: Standard of the mean.

In caecal digesta, supplementation with BPP had a significant effect on *Salmonella* Gallinarum counts on days 10 (*P* = 0.032) and day 21 (*P* = 0.02) (Table 4). On day 10, the SGI group had the highest *Salmonella* Gallinarum counts, indicative of intestinal colonization; however, no significant differences were observed among SGI, BPP-1, BPP-2, and CIPRO groups, indicating a comparable reduction pattern among the treated groups. On day 14, no significant differences were observed in *Salmonella* Gallinarum counts among the groups (*P* = 0.092). By day 21, only the SGI group maintained significantly higher bacterial counts than all other groups, including NC, while BPP-supplemented and CIPRO-treated birds showed markedly reduced counts, demonstrating effective suppression of intestinal colonization. Among all challenged treatments, the CIPRO group exhibited the greatest reduction, effectively clearing the infection by day 21 (*P* = 0.002).

### Histopathological assessment analysis

**Fig. 3:**
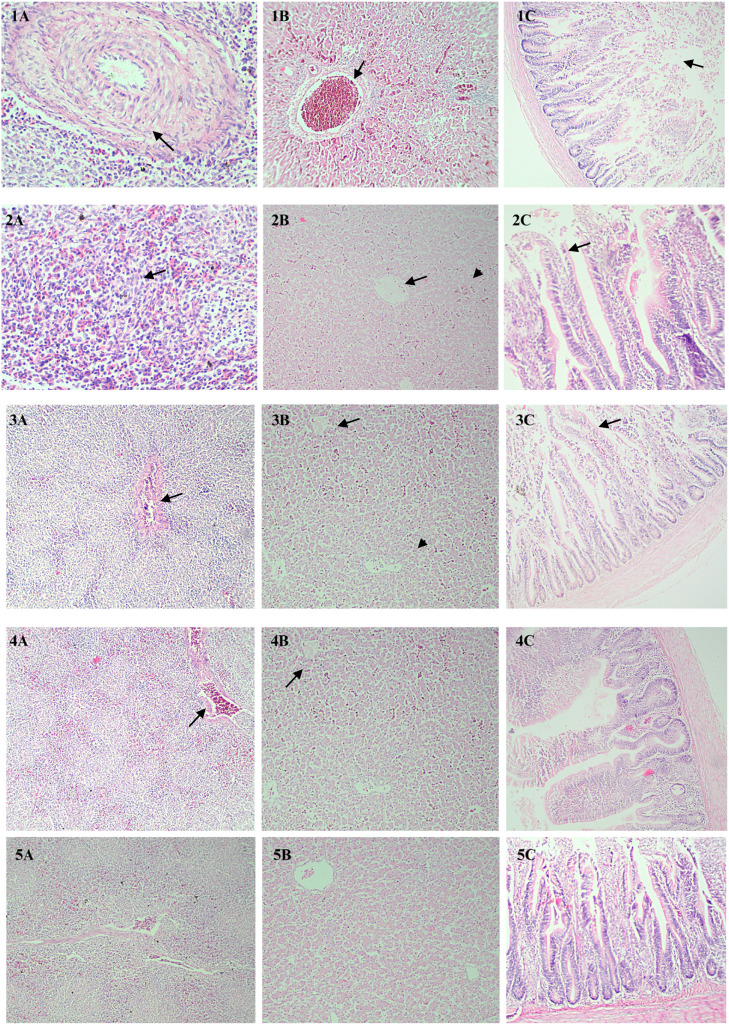
**Photomicrographs of spleen, liver and intestinal tissue sections of broiler chicks post 21-days of experimentally induced *Salmonella* Gallinarum Infected birds (SGI).***The right panel displays splenic sections, the middle panel shows hepatic sections, and the left panel presents intestinal sections. Experimental groups include: Non-challenged control (5A, 5B&5C), Salmonella* Gallinarum *experimentally challenged group (1A, 1B&1C), Group supplemented with a basic diet* + 2 *% banana peel extract powder (BPP-1) (2A, 2B&2C), Group fed with a basic diet* + 4 *% banana peel extract powder (BPP-2) (3A, 3B&3C) and group treated with Ciprofloxacin (CIPRO) (4A, 4B&4C). Different arrows show: (1A) fibrinoid necrosis of splenic vessel, (1B) hepatic portal congestion, (1C) disrupted villi, (2A) focal necrosis and lymphocytic infiltration, (2B) portal vein congestion, (2C) mild villous degeneration, (3A) moderate fibrosis of spleen, (3B) Mild congestion of hepatic portal vein and focal necrosis, (3C) mild degeneration of intestinal villi, (4A) mild splenic blood vessel congestion and (4B) Mild congestion of hepatic portal vein. Arrow heads indicate (2B) focal necrosis of hepatic tirade and (3C) mild coagulative necrosis (10X).*

Upon histopathological assessment, spleen, liver, and intestinal tissue sections from the SGI birds revealed marked fibrinoid necrosis of the splenic blood vessels (Fig. 3(1A) arrows), severe congestion of the hepatic portal vein (Fig. 3(1B) arrows). In caeca, moderate disruption of villi along with derangement of the brush border with moderate tissue degeneration (Fig. 3(1C) arrows). This histopathological pattern is indicative of *Salmonella* Gallinarum-induced a progressive pathological process that noticeably compromises the splenic, hepatic, and intestinal functions. In contrast, broiler birds in the BPP-1 group showed mild to moderate fibrosis in the spleen (Fig. 3(2A) arrows), mild congestion with few areas of focal coagulative necrosis in the hepatic tissue sections (Fig. 3(2B) arrows), and a mild degeneration of the intestinal villi (Fig. 3(2C) arrows). On the other hand, birds in the BPP-2 group exhibited mild congestion in the splenic blood vessels and hepatic portal vein (Figs. 3A and 3B) arrows) with fewer areas showing marked regenerative changes in the intestinal villi (Fig. 3C). Mild congestion of hepatic and splenic blood vessels was the only lesion observed in Ciprofloxacin-treated group (Figs. 3 (4B and 4C) arrows). In contrast, histopathological sections from the control group showed an intact architecture of the spleen and liver tissue (Fig. 3 (5A and 5B)), and a normal brush border of the intestinal villi (Fig. 3(5C)).

### Growth performance

On day 7, weight gain, feed intake and FCR showed no statistically significant differences among treatment groups (*P* = 0.81). However, by days 14 and 21, supplementation with BPP had a significant effect on the growth performance of birds (Table 5). Birds in the BPP-2 groups showed significantly higher weight gain on day 14 (309 g) and day 21 (575 g) compared to the SGI and BPP-1 groups (*P* < 0.01) but did not differ from the NC and CIPRO groups. Similar findings were observed for FCR, reflecting optimal nutrient consumption and better feed efficiency in the BPP-2 group. In contrast, on days 14 and 21, the SGI group had the lowest weight gain (*P* < 0.01) and the highest FCR (*P* < 0.01), showing that the infection significantly impaired growth performance and reduced feed efficiency. In terms of feed intake, no statistically significant differences were observed among treatment groups on days 7 (*P* = 0.785) and 14 (*P* = 0.82). However, by day 21, SGI birds had highest feed intake (*P* = 0.017) than other treatment groups.

**Table 5:** Effect of Banana peel extract powder supplementation on weight gain, FCR and Feed intake of broiler chickens experimentally challenged with *Salmonella* Gallinarum.

GROUP	Weight gain(g)				FCR			Feed intake(g)		
	Day 7	Day 14	Day 21	Day 7		Day 14	Day 21	Day 7	Day 14	Day 21
NC	147.5	320.09^a^	606.33^a^	2.6		1.92^a^	1.283^b^	335.1	596	1221^b^
SGI	147.6	299.21^d^	539.67^d^	2.4		3.213^e^	1.850^a^	334.6	591.3	1229^a^
BPP-1	146.8	301.12^c^	562.67^c^	2.5		2.717^d^	1.515^a^^b^	331.8	594.4	1216^b^
BPP-2	144.4	309.15^b^	575.29^b^	2.55		2.473^c^	1.475^c^	338.4	599.4	1214^b^
CIPRO	148.1	311.05^a^	593.97^a^	2.62		2.183^b^	1.287^b^	332	592.3	1215^b^
p-value	0.81	<.001	<.001	0.85		<.001	<.001	0.785	0.82	0.017
SEM	3.24	2.115	1.719	0.0353		0.0294	0.0225	4.11	5.22	3.19

NC = Non-challenge control group; SGI = *Salmonella* Gallinarum challenge group; BPP-1 = group fed basic diet + 2 % banana peel extract powder + *Salmonella* Gallinarum challenge; BPP-2= group fed basic diet + 4 % banana peel extract powder with *Salmonella* Gallinarum challenge; CIPRO= Ciprofloxacin with *Salmonella* Gallinarum challenge.

a-e Superscripts on different means within a column differ significantly (*P* < 0.05) SEM: Standard of the mean.

**Table 6:** Gas chromatography–mass spectrometry phytochemicals detected in Banana peel extract powder.

Peak Number	RT (min)*	Name of compound	% of total	Biological Activities
1	2.341	2-Pentanol, acetate	2.24	Immunomodulatory, Anti-inflammatory, Antioxidant, (Chóez-Guaranda, et al., 2024) and Antimicrobial (Bukvicki, et al., 2012).
2	17.271	n-Hexadecenoic acid	10.95	Immunomodulatory (Asogwa, et al., 2023) Antioxidant (Purushothaman, et al., 2024), Antimicrobial (Ganesan, et al., 2024), Anti-inflammatory (Purushothaman, et al., 2025).
3	18.447	9,12-Octadecadienoic acid	1.34	Immunomodulatory (Winanta, et al., 2025), Antioxidant (Boora, et al., 2023), Antimicrobial (Qureshi, et al., 2020) and Anti-inflammatory (Boora et al., 2023).
4	18.531	9-Octadecenoic acid, methyl ester	2.04	Immunomodulatory (Sanagala et al., 2024), Antioxidant (Hussein and Hamad, 2020), Antimicrobial (Khalid, et al., 2024) and Anti-inflammatory (Ogbuagu, et al., 2021).
5	18.92	Oleic acid	44.82	Immunomodulatory (Coniglio, et al., 2023), Antioxidant (Kazlauskienė, et al., 2021), Antimicrobial (Charlet, et al., 2022).
6	19.126	9-Octadecenoic acid, (E)-	6.32	Immunomodulatory (Asogwa, et al., 2023), Antioxidant (Hussein and Hamad, 2020).
7	20.999	tert-Hexadecanethiol	1.29	Immunomodulatory, Antioxidant (Elshaarawy, et al., 2023) and Antimicrobial (Sabira, et al., 2022).
8	21.524	2-Methyl-Z,Z-3,13-octadecadienol	1.25	Immunomodulatory (Asogwa, et al., 2023), Antioxidant, Antimicrobial and Anti-inflammatory (Srief, et al., 2024).
9	21.584	Cyclopropaneoctanal, 2-octyl-	3.51	Immunomodulatory, Antioxidant, Antimicrobial and Anti-inflammatory (Srief et al., 2024).
10	21.811	1-Heptadecene	1.44	Immunomodulatory, Antioxidant (Hamidi, et al., 2012) and Antimicrobial (Srief et al., 2024).
11	23.158	9-Octadecenoic acid (Z)-, 2,3-di	15.41	Immunomodulatory, Antioxidant, Antimicrobial (Mathew and Mathew, 2023) Anti-inflammatory (Srief et al., 2024).
12	23.263	2-Methyl-Z,Z-3,13-octadecadienol	3.73	Immunomodulatory, Antioxidant, Antimicrobial (Srief et al., 2024) Antimicrobial and Anti-inflammatory (Singh and Kwalyambumu, 2024).

⁎ Retention time (RT).

## Discussion

The therapeutic potential of BPP observed in this study can be attributed to its rich phytochemical profile, which included phenolics, flavonoids, tannins, saponins, and glycosides (Table 1). These bioactive compounds are well-documented for their antioxidant, antimicrobial, and immunomodulatory properties (Someya et al., 2002). Particularly notable was the detection of gallic acid via HPLC analysis, a phenolic compound with established efficacy against Gram-negative bacteria, including *Salmonella typhimurium* (Devatkal et al., 2014; Liu et al., 2025). The presence of gallocatecthins, which occur at significantly higher concentrations in banana peels (158 mg/100 g) compared to pulp (29.6 mg/100 g) (Pramote et al., 2018), likely contributed to the antioxidant and antimicrobial effects observed in our study. These phytochemicals exert their antibacterial effects through various mechanisms, such as disruption of bacterial cell membranes, inhibition of metabolic pathways, and interference with bacterial DNA synthesis (Khameneh et al., 2021), which may explain the reduced Salmonella Gallinarum burden in BPP-supplemented birds. Earlier studies (Shivaprasad, 2000; Nazir et al., 2024) have reported similar pathological lesions with *Salmonella* Gallinarum infection, lesions being more pronounced in broiler chicken at their growing stage (2-3 weeks). In the present study, necropsy revealed that lesion severity peaked during the second week post-challenge (days 10-14), with SGI birds showing hemorrhagic liver, enteritis, caecal ballooning, and splenomegaly, typical lesions of systemic salmonellosis. This acute phase coincided with peak IL-6 expression and highest bacterial load, suggesting a critical stage during which immune modulation is most beneficial. The finding that lesion severity reduced by week three, despite persistent infection in the SGI group, indicates either activation of the immune response or bacterial persistence in a less virulent state. Importantly, the timing and nature of these lesions align with previous reports in broiler chickens at 2-3 weeks of age (Shivaprasad, 2000; Saleem et al., 2022), confirming that this developmental stage represents a vulnerable period for *Salmonella* Gallinarum infection. Supplementation with BPP, particularly at 4 %, significantly reduced these pathological changes, suggesting effective immune modulation during this critical period.

Consistent with our result showing IL-6 peak at day 14 post-challenge, reflects the acute inflammatory response to systemic Salmonella infection. While IL-6 plays an essential role in early pathogen defense through neutrophil recruitment and acute phase protein synthesis (Tanaka et al., 2018), excessive or prolonged elevation can exacerbate tissue damage, as observed in current histopathological findings. Supplementation with BPP effectively mitigated this hyperinflammatory response, with both BPP-1 and BPP-2 groups. This modulation is critical as it suggests BPP does not simply suppress immunity but rather prevents excessive inflammation that could compromise tissue integrity. The mechanism likely involves the anti-inflammatory properties of tannins, saponins, and phenolic compounds present in banana peel, which have been shown to inhibit pro-inflammatory cytokine production (Zaki Ibrahim et al., 2022). Particularly, the CIPRO group showed similar IL-6 downregulation, confirming that effective bacterial control, whether through antimicrobial compounds or antibiotics, reduces the inflammatory stimulus. However, BPP offers the advantage of simultaneously providing immunomodulation without contributing to antibiotic resistance.

SGI group showed markedly reduced MHC class IIβ expression, reaching at its peak at day 14, along with the peak time point of *Salmonella* Gallinarum. This downregulation reflects a key virulence strategy of *Salmonella* Gallinarum: immune evasion through disruption of antigen presentation. By invading macrophages and utilizing Salmonella Pathogenicity Islands (SPI-2, SPI-3) and PhoPQ systems, the pathogen effectively dismantles MHC class II expression, preventing CD4+ T cell activation and crippling adaptive immunity (Bernal-Bayard and Ramos-Morales, 2018; Zhou et al., 2023). This phenomenon has been consistently reported in *Salmonella* Gallinarum-challenged chickens (Kaiser et al., 2022; Zhang et al., 2025). Supplementation with BPP particularly (4 %) helped to decrease the higher MHC class IIβ expression, representing its possible immunoprotective mechanism. By day 14, BPP-2 birds showed significantly higher MHC class IIβ expression than SGI birds in both liver and caecal tissues, indicating enhanced macrophage activation and antigen presentation capacity. This restoration of adaptive immunity likely contributed to the improved bacterial clearance observed in BPP-supplemented groups. Bioactive compounds in banana peel, particularly tannins and lectins, may facilitate this effect by supporting macrophage function and MHC class II stability (Chueh et al., 2019; Parker and Kaufman, 2017). The practical implication is clear: BPP not only combats bacterial pathogens directly but also enhance effective adaptive immune responses of the birds.

The present study showed that highest percentage of *Salmonella* Gallinarum (80 %) in the caecal digesta of the birds on 10 and 14^th^ day following challenge with *Salmonella* Gallinarum. Supplementation of BPP significantly reduce the *Salmonella* Gallinarum in the intestine of broiler chickens. Higher concentrations of SG were recovered from the liver on day 21 (Table 4). Similar to our finding, Haider et al. (2014) recovered the highest concentrations of *Salmonella* from the liver and lowest from GIT, particularly the crop.

In SGI birds, bacterial counts were highest in caecal digesta on days 10 and 14, reflecting successful intestinal colonization. Supplementation with BPP, particularly at the 4 % level (BPP-2), significantly reduced bacterial burden in both tissues, provided direct evidence antibacterial activity of BPP. This bacterial reduction was most pronounced in caecal digesta where the extract would have direct contact with the pathogen. This dose-dependent reduction (BPP-2 > BPP-1) may suggests that phytochemical concentration is critical for its antimicrobial efficacy.

The antibacterial mechanism likely involves multiple pathways. Recent work has demonstrated that banana peel-derived compounds disrupt membrane integrity and impair core metabolism in Gram-negative bacteria such as Pseudomonas fluorescens (Meng et al., 2025). Given the structural similarities between Pseudomonas and Salmonella as Gram-negative organisms, comparable mechanisms may explain our findings. Additionally, the phenolic compounds, particularly gallic acid identified in HPLC analysis, have established anti-Salmonella activity (Liu et al., 2025). The concurrent reduction in bacterial load, decreased inflammatory markers (IL-6), and enhanced adaptive immunity (MHC class IIβ) in BPP-supplemented birds shows a synergistic effect: BPP both directly inhibits pathogen growth and supports immune-mediated clearance. Interestingly, bacterial counts showed non-significant differences among groups on day 14 in liver tissue, possibly indicating either effective early clearance by BPP or activation of the natural immune defenses of the host. By day 21, CIPRO-treated birds had completely eliminated detectable bacteria, confirming antibiotic efficacy, but BPP-2 birds also showed substantial reductions approaching those of medicated birds.

The histopathological findings supports protective effects of supplementation of BPP at the tissue level. In SGI birds, severe pathological changes particularly hepatic necrosis, splenic lymphoid depletion, and intestinal villous atrophy shows *Salmonella* Gallinarum capacity to compromise multiple organ (Farhat et al., 2023; Hu et al., 2024). Supplementation with BPP-1 group showed moderate improvement whereas BPP-2 group exhibited remarkable tissue preservation therefore mitigating pathological lesions as a result of *Salmonella* Gallinarum challenge This restoration of intestinal architecture is especially significant because the gut not only serves the primary site of Salmonella invasion but also as a critical component of mucosal immunity. The regenerative capacity observed with BPP-2 likely results from its anti-inflammatory properties (reduced IL-6), antioxidant activity (phenolic scavenging reactive oxygen species), and tissue repair stimulation by compounds such as saponins and flavonoids (Savitri et al., 2022; Rukyat et al., 2025). While the CIPRO group also showed minimal pathological changes, the use of BPP offers distinct advantages. With prolonged use antibiotics there is potential risks of resistance development and potential hepato-renal toxicity (Yuan et al., 2023; Abdulmawjood et al., 2024), plant-derived supplements provide antimicrobial activity simultaneously with tissue repair and better immunity.

The impact of *Salmonella* Gallinarum infection on growth performance of SGI group was manifested by reduced feed intake, decreased weight gain, and poor FCR. Supplementation of BPP, particularly at 4 %, significantly ameliorated these negative effects. This growth promotion likely results from multiple mechanisms: reduced bacterial load decreasing the metabolic burden, modulated inflammation (lower IL-6), improved gut health leading to better nutrient absorption, and the nutritional contribution of banana peel itself, which contains 4-10 % crude protein (Hashim et al., 2023). These findings align with previous reports that dietary inclusion of banana peel improves body weight and FCR in both poultry and ruminants (Xue et al., 2020; Ansari et al., 2023; Saeed et al., 2025).

A consistent finding across all measured parameters was the superior performance of 4 % BPP (BPP-2) compared to 2 % BPP (BPP-1), demonstrating clear dose-dependent effects. This dose-response may suggest that the concentration of bioactive phytochemicals is critical for therapeutic efficacy, consistent with established principles of phytochemical antimicrobial activity (Tsuchiya, 2015).

## Conclusions

These findings suggest that dietary supplementation of BPP, particularly at 4 % modulates immune function, improves gut morphology, and enhance growth performance in broilers challenged with *Salmonella* Gallinarum. Further research is needed to evaluate long-term safety, cost-effectiveness and practical application of BPP in commercial poultry.

## Funding statement

The authors extend their appreciation to the ongoing Research Funding Program (ORF-2025-552) King Saud University, Riyadh, Saud Arabia.

## Publishing consent

The authors extend their consent to publish.

## CRediT authorship contribution statement

**Gulbeena Saleem:** Writing – review & editing, Writing – original draft, Supervision, Resources, Project administration, Methodology, Investigation, Formal analysis, Conceptualization. **Nabiha Fatima:** Writing – review & editing, Writing – original draft, Visualization, Methodology, Data curation. **Maryam Tariq:** Writing – review & editing, Writing – original draft, Methodology. **Asad Ullah:** Writing – review & editing, Writing – original draft, Methodology. **Bushra N. Khan:** Resources, Investigation. **Sadia Chaman:** Validation, Project administration. **Mostafa A. Abdel-Maksoud:** Funding acquisition. **Abdulaziz Alamri:** Writing – original draft, Funding acquisition. **Aljawharah F. Alabbad:** Resources, Funding acquisition. **Fatma Sh. Kalmosh:** Writing – review & editing, Resources.

## Disclosures

The authors declare that they have no known competing financial interests or personal relationships that could have appeared to influence the work reported in this paper.
